# Scaly-tail organ enhances static stability during Pel’s scaly-tailed flying squirrels’ arboreal locomotion

**DOI:** 10.1098/rsif.2024.0937

**Published:** 2025-06-25

**Authors:** Andrew K. Schulz, Mrudul Chellapurath, Pranav C. Khandelwal, SeyedReza Rezaei, Stefan Merker, Ardian Jusufi

**Affiliations:** ^1^ Locomotion in Biorobotic and Somatic Systems Group, Max Planck Institute for Intelligent Systems, Stuttgart, Germany; ^2^ Department of Zoology, State Museum of Natural History Stuttgart, Stuttgart, Germany; ^3^ Engineering Sciences Department, Empa Swiss Federal Laboratories for Materials Science and Technology, Dübendorf, Switzerland

**Keywords:** gliding, bio-inspiration, friction, *Anomularidae*, tail, mammal

## Abstract

Scaly-tailed squirrels (Anomaluridae) are one of the least studied mammalian families. Their name is due to a peculiar and unique scaly-tail organ extruding from the caudal vertebra that has been predicted to help reduce skidding. This study investigates the function of the scaly-tail organ found in *Anomalurus pelii*, investigating its potential role in enhancing arboreal locomotion. As these animals glide from tree to tree in a habitat abundant with smooth-bark trees, we hypothesize that the scaly-tail organ assists with friction enhancement in their native smooth-bark habitat. Through a combination of analyses using mathematical and physical models for experimental validation, we explore whether the scaly-tail organ could improve the sliding and pitching stability during perching. Our experimental results showed that the scaly-tail organ can act as a skid-reduction mechanism by enhancing substrate engagement on intermediate roughness substrates by 58%. Mathematical models showed the scaly-tail organ enhances static pitch stability by acting as an additional support point. Our model showed that the scaly-tailed squirrel can reach up to 82.5° inclination without claw force; however, without scales, it reduces to 79.6°. Overall, this research highlights the functional significance of scaly-tail organs in adaptations in scaly-tailed flying squirrels and contributes to our understanding of their locomotion strategies and environmental stresses. Our study also provides insights into innovative locomotion mechanisms for robots operating in arboreal environments.

## 1. Introduction

Arboreal environments are highly three-dimensional discontinuous structures in which animals move horizontally and vertically. They do so by performing behaviours including climbing, jumping, perching, hanging, descending and even gliding [1] while interacting with various substrates, from rough tree barks or branches to relatively smooth, slippery surfaces. The variability of surface texture and composition necessitates different behavioural and morphological adaptations to maintain traction and stability and not fall while performing these behaviours [2]. Large body-sized animals like primates use long appendages to grasp and hold tree branches and bridge gaps [3]. Snakes use their elongated bodies to generate sufficient muscular force to wrap and grip the arboreal surface [4]. As body size decreases, certain morphological adaptations such as adhesive pads, claws and spines tend to become more mechanically effective and more prevalent, while other adaptations like prehensile tails are found across various body sizes [5]. These adaptations are seen in animals spanning approximately seven orders of magnitude in body mass, from mites to lizards [6–8].

Morphological adaptations such as claws and adhesive pads have been extensively studied in lizards and have been shown to be an effective way to grip the arboreal substrate and generate substrate reaction forces during climbing [9]. The insights from these studies have also been applied to bio-inspired robots, enabling them to scale inclines and vertical substrates [10]. Additionally, claws and adhesive pads [8] can be complemented by the use of the tail, which can act as a fifth point of contact between the substrate and the body. For example, chameleons exhibit a prehensile tail, which can be coiled around a perch during slow climbing to act as an anchor point while crossing gaps [11]. Also the distal tail tips of birds can be used to support them during climbing, which is achieved through geometry and not material shifts [12]. Treecreepers can use the tail tip anchorage with specialized feathers for station holding during large impulse dissipation as the beak causes perforation in the tree trunk [13].

When it comes to dynamic vertical locomotion, geckos were found to use a tail reflex to reject perturbations, with reduced climbing performance documented in tailless animals [14]. Further substantiating the hypothesis of the function of contact tails in disturbance rejection during rapid vertical running, the tails of highly arboreal lizards were seen to be in contact during climbing of rough substrates [15]. The tails had keeled subcaudal scales with spines pointing distally that were hypothesized to aid in disturbance rejection during climbing on rough substrates. Additionally, experiments on individual scales showed that the scale could sustain forces to support at least the body mass of the lizard in three lizard species [15].

Gliding geckos can press their tail against the substrate to prevent pitch back and falling during landing on trees [15], and the impact dynamics experimentally validated with a soft gecko-inspired robot landing using an active tail reflex [16]. Active tail movement can also facilitate mid-air torso reorientation by appendage inertia, as has been demonstrated in lizards [14,15,17,18] and seen in squirrels [19]. The role of the active tail robot’s [16] back and tail stiffness was evaluated with passive, unactuated, at-scale physical models of perching geckos [20]. These studies highlight the role of the tail as an appendage with functions critical to locomotion performance. Interestingly, as seen in highly arboreal lizards [15], the tails of some arboreal animals possess specialized morphological features such as spines and scales, which can provide additional mechanical support during climbing [15], or potentially during perching [21]. However, their role in perching remains under-explored. Robot locomotion experiments demonstrate that by combining functional materials with engineering principles, additional benefits can be achieved, including generalist locomotion or reduction of energy cost [22,23].

**Figure 1.**
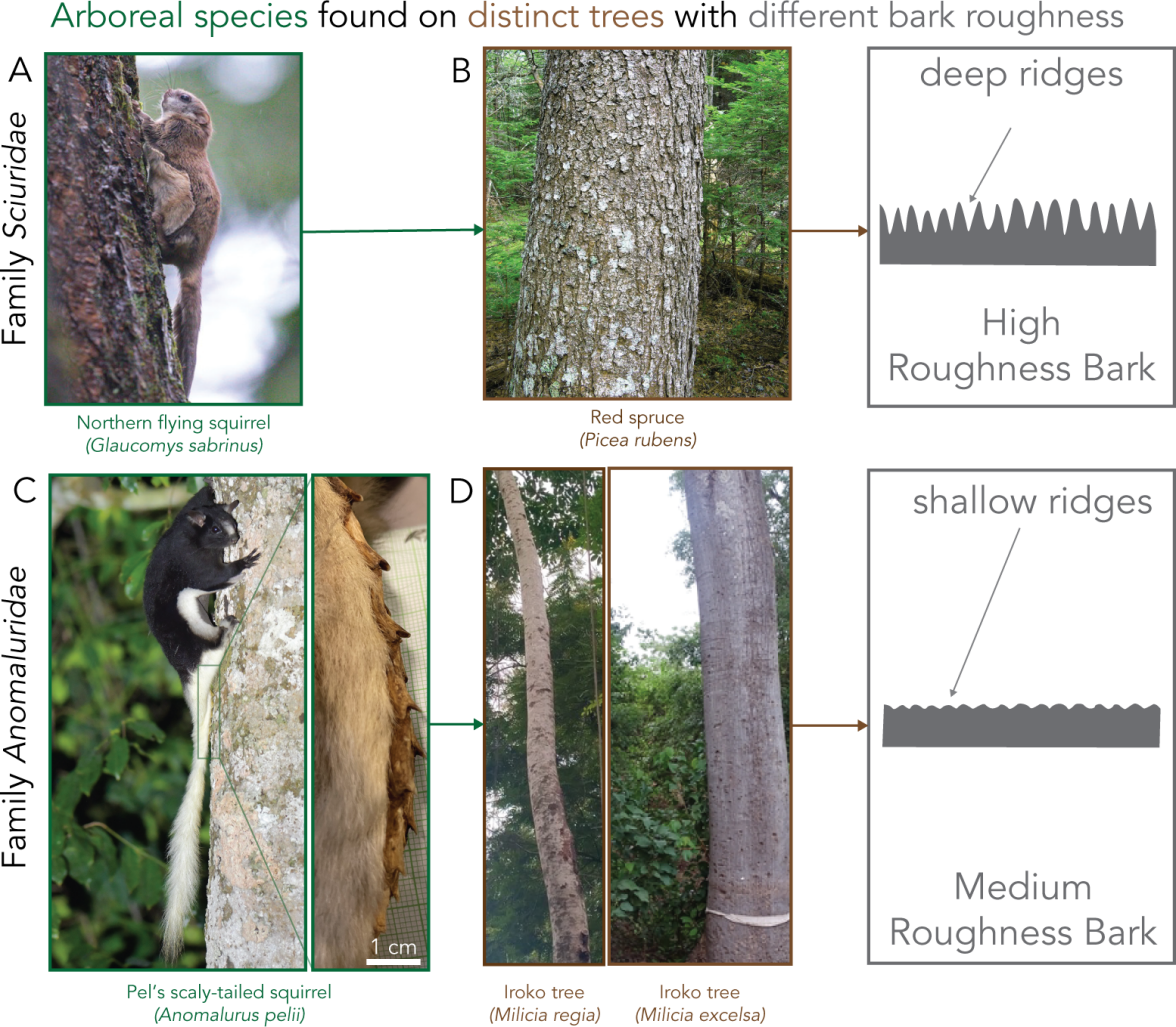
Arboreal mammalian species with native trees used for climbing. (A) Image of northern flying squirrel (*Glaucomys sabrinus*) climbing a tree, photo taken by photospaul. (B) Image of red spruce, commonly climbed by the northern flying squirrel, with a schematic showing the deep ridges from the scaled surface of the spruce tree. (C) Image of the Pel’s scaly-tailed squirrel (*Anomalurus pelii*) we examine in this study taken by pfaucher with an inset of a museum specimen from the State Museum of Natural History Stuttgart showing a scaly organ on the ventral caudal portion of the tail. (D) Two common trees found in their native habitat of West Africa, the *Milicia regia* and *Milicia excelsa*. As the schematic shows, these are described as smooth-bark trees with smooth surfaces and small ridges caused by creases along the tree. Photos taken by agboola and Sadamtoro. Photos are taken from iNaturalist, all under creative license CC BY-NC.

In this study, we investigate the role of one such tail adaptation found in the elusive scaly-tailed squirrels, a family of rodents native to Africa. These squirrels have a unique tail adaptation called the ‘scaly-tail organ’ with scales that protrude from the caudal spine bones out of the skin (figure 1; electronic supplementary material, figure S1) [21]. This adaptation is shared across all species in the family Anomaluridae, which include *Anomalurus pusillus*, *A. beecrofti*, *A. derbianus*, *A. pelii, Idiurus macrotis* and *I. zenkeri* [21]. These species exhibit a patagium, enabling them to glide and traverse their habitat, unlike the closely related flightless taxon *Zenkerella insignis* that lacks a patagium [24]. Still, Anomaluridae are more closely related to non-gliding rodents than they are to flying squirrels, which belong to the family Sciuridae (figure 1, electronic supplementary material, table S1). A distinctive feature of the scaly-tailed squirrels’ habitat, specifically that of *Anomalurus pelii* or Pel’s scaly-tailed squirrel, is a high abundance of trees that are often referred to as ‘smooth-barked’ trees [25] shown in figure 1D. These trees have barks that are much smoother than tree barks found in habitats of flying squirrels that lack the scaly-tail adaptation, such as the Mentawi flying squirrel (*Iomys sipora*), woolly flying squirrel (*Eupetaurus cinereus*) and Sipora flying squirrel (*Hylopetes sipora*) [26]. The unique tail adaptation in the Pel’s scaly-tailed squirrel habitat has led to the hypothesis that the squirrel’s scaly-tail organ has evolved as a skid reduction mechanism and to support body weight while perching on the tree trunk [24,27]. However, these hypotheses are derived from field observations and have not been supported by experiments [28–31].

Our study addresses these hypotheses and further investigates the scaly-tail organ’s role in enhancing the squirrel’s perching capabilities. We do this by characterizing the three-dimensional shape of the Pel’s scaly-tailed squirrel claw and scaly-tail organ, and using that to perform a static stability analysis of the squirrel’s perching behaviour. We analyse two types of static instability that any animal/object can encounter on an inclined surface: sliding and overturning (specifically, pitching). Sliding happens when the component of gravitational force is greater than the frictional resistance provided by the points of contact with the substrate. Pitching can take place when the horizontal projection of the squirrel’s centre of mass (COM) moves out of the support polygon (defined as the convex polygon formed by connecting the supports on the substrate) [32].

Using a 3D-printed physical model of the scaly-tail organ and claws, we investigated the sliding stability with experiments on an angular varying sandpaper ramp, which has been demonstrated to mimic the roughness of bark substrates [33]. We proceeded with mathematical simulations to test the impact of the scaly-tail organ on pitching stability during climbing or perching [15,16]. Overall, the sliding stability analysis tested the hypothesis that the scaly-tail organ acts as a skid reduction mechanism for the squirrel and can support body weight on ‘smooth’ bark-like surfaces compared with the squirrel model without the scaly-tail organ. Specifically, we predicted that the scaly-tail organ would show similar frictional characteristics as the claws on extremely smooth substrates but significantly improve substrate engagement on intermediate roughness substrates. Furthermore, we hypothesized that the scaly-tail organ size is adapted to maximize the perch angle, and a smaller or larger scaly-tail organ size would reduce the perch angle, probably due to poorer engagement of the scales with the substrate.

The pitching instability was investigated using a mathematical model that simulated different scaly-tail organ sizes and the squirrel’s COM position to understand how the support polygon formed by the fore-hindlimb claws and the scaly-tail organ could influence pitching, as has been shown in geckos, in the kick-stand response during climbing [14,15] or the fall arrest response during perching [16]. Based on this analytical approach, we hypothesized that the scaly-tail organ enhances the overturning stability of the squirrel while perching by increasing the area of the support polygon within which the projection of COM lies compared with a support polygon formed by the fore and hindlimb claws only. The presence of scales is critical in this case because at steeper angles of perching, the tail without the spines would slip and would not be able to act as a support point. Specifically, we would observe a steeper incline threshold for pitching for support polygons formed with the scaly-tail organ compared with the polygon with only four claws.

## 2. Material and methods

### 2.1. Squirrel morphological data

**Figure 2.**
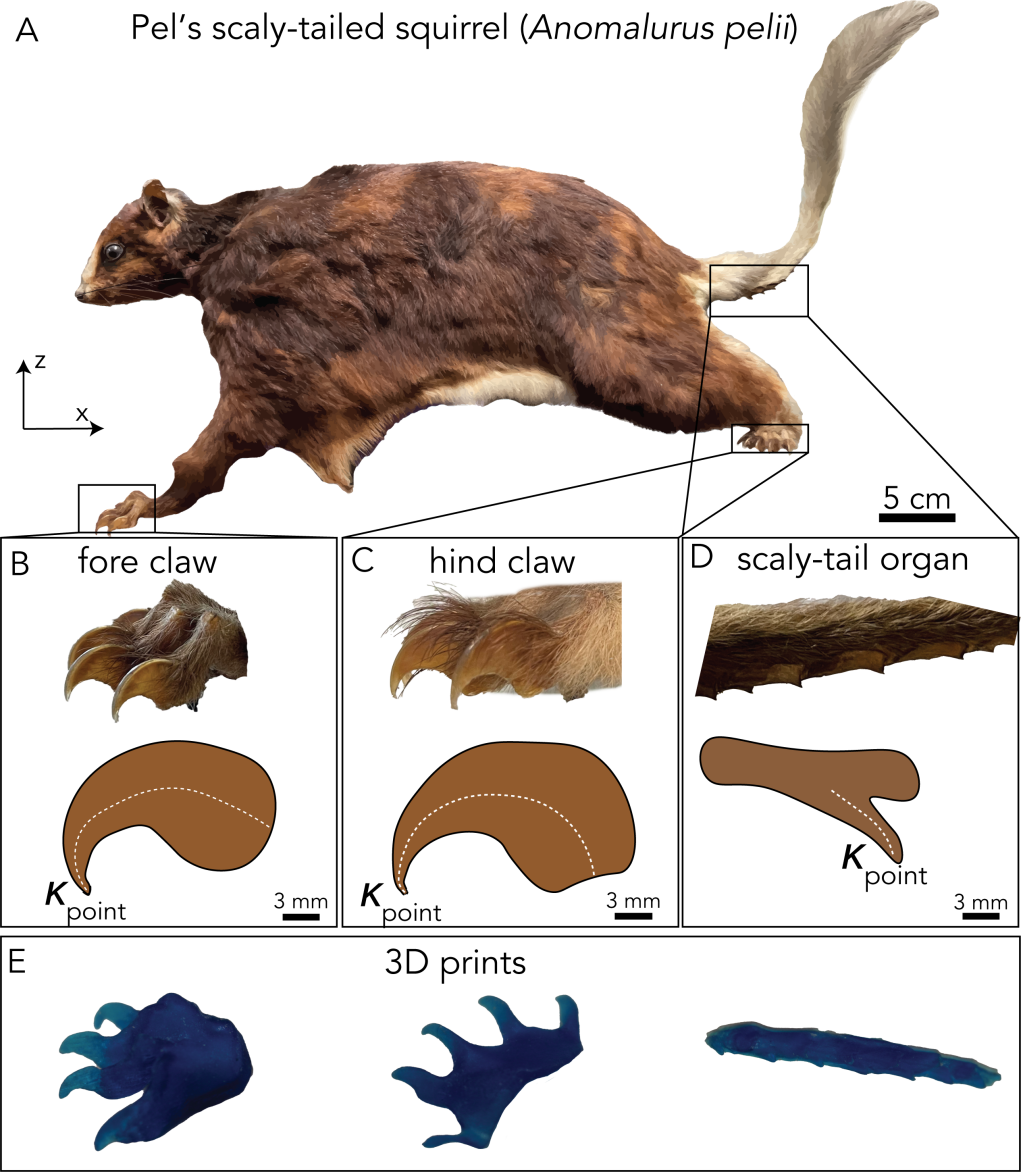
Three-dimensional scanned portions of Pel’s scaly-tailed squirrel. (A) Mounted museum specimen (SMNS-Z-MAM-001781) of *Anomalurus pelii* used for three-dimensional scanning, (B–D) fore claw, hind claw and scaly organ of the squirrel and a side view of the curvature and point curvature of each keratinized section of the squirrel. (E) 3D-printed fore claw, hind claw and scaly organ from scans of the mounted specimen.

Morphological data were taken from a single specimen of Pel’s scaly-tailed squirrel, also known as Pel’s scaly squirrel (*Anomalurus pelii*), at the State Museum of Natural History Stuttgart (SMNS) in November 2022. The specimen skin (SMNS-Z-MAM-001781), which was collected in Ghana in 1881, is shown in figure 2A.

Photographs of the entire squirrel and close-ups of the claws and scaly tail were taken against a 1 × 1 cm graph sheet as shown in figure 2A–D and electronic supplementary material, figure S2. Post photographs, the mounted Pel’s scaly-tailed squirrel was three-dimensionally scanned using a handheld scanner (Artec Space Spyder 3D Scanner) by placing it on a plastic turntable. Scanning was performed at eight frames per second (fps) at a distance ranging from 18 to 30 cm away from the specimen. Six scans were performed, including two scans of each site: the scaly-tail organ, fore claw and hind claw. Due to the staging of the mounted specimen in the museum collection, the right fore paw and left hind paw were partially obstructed and could not be scanned to complete a three-dimensional closed object. Therefore, the other hind claw and fore claw were mirrored to complete the scanned results.

The Artec 3D scans traced both the geometry and texture. The total scanned surfaces differed between the fore claw, hind claw and the tail organ, as the surface size varies based on the sample volume scanned. Scanned samples averaged 800 surfaces for the claws and 1200 for the caudal scaly-tail organ.

The scans taken of each structure were imported into Artec 3D Professional from Artec Studio 18, and the raw scans were cropped only to include the structures of interest (i.e. claws and scaly-tail organ). The scans were converted into three-dimensional mesh structures using the Artec software’s automated tool. The minor irregularities in the three-dimensional model, like small holes, were manually filled and fitted with the default spline interpolation provided in the software. Moreover, smoothing allowed removing any spikes in the scan caused by the hair on the sample, leading to a solid structure for 3D printing (figure 2E).

Finally, the three-dimensional scans were used to take measurements of the fore claw, hind claw and the scaly-tailed organ (figure 2B–D). The photographs were used to measure the body and appendage length reported in the supplement (electronic supplementary material, figure S3, table S3).

The curvature of the claws/scale tips was taken as the two-dimensional curvature based on an arc length of the line surrounding the structure. We denote this arc length function as *y*(*x*), which is the function of a position, *x*. We can find the curvature of this arc length by using the function in the curvature equation given by


(2.1) $$\kappa =\frac{y^{{\;}^{\prime \prime }}\left(x\right)}{{\left(1+y^{\prime }{\left(x\right)}^{2}\right)}^{3/2}},$$

where *y*(*x*) is a function of the centre-line of curvature. We took the curvature along the tips of each of the claws/spines to calculate the ball tip curvature as seen in figure 2B–D.

### 2.2. Sliding stability analysis

#### 2.2.1. Squirrel physical model creation

The solid three-dimensional models generated using the Artec software were printed (figure 2E) on a Stratasys J835 PolyJet 3D printer using Vero transparent blue printing material. This material has an approximate tensile strength of 50 to 60 MPa and a modulus of elasticity of 2000 to 3000 MPa [34]. The scaly-tail organ is keratinous [21], which is similar to that of claw- and horn-like keratin tissues between 1500 and 4000 MPa with a tensile strength of around 30−77 MPa [35]. To create a physical model for frictional tests, we geometrically scaled down all morphological data of the Pel’s scaly-tailed squirrel to half the size of the scanned squirrel (electronic supplementary material, table S1, figure S2). The model’s mass was proportionately scaled down using the scaling law (*m* ∝ *l*^3^), which made the entirety of the physical model approximately 0.5 kg.

**Figure 3.**
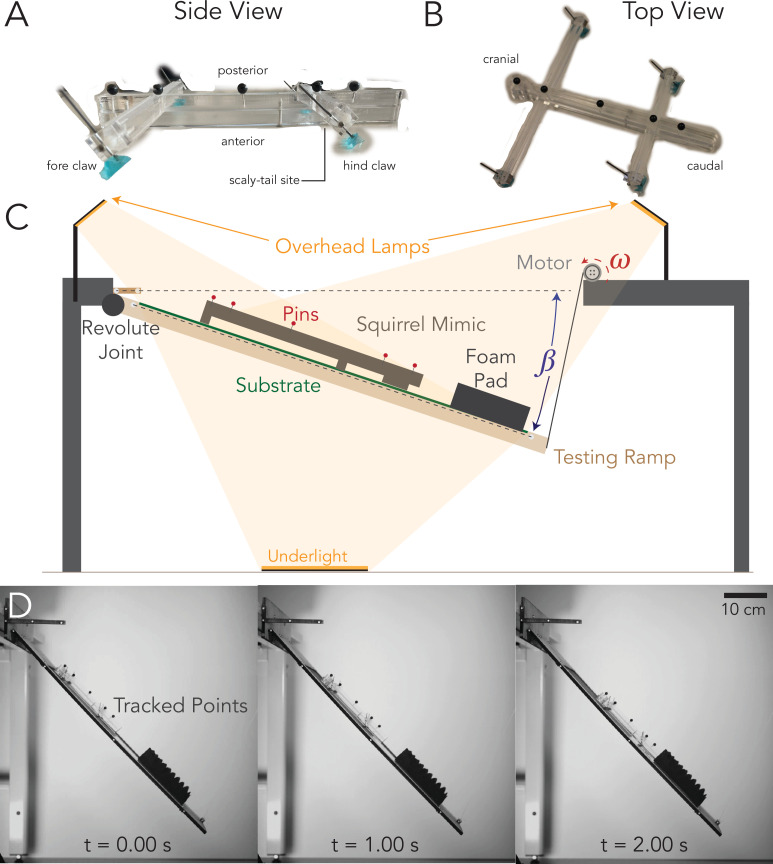
(A) Side view of scaly squirrel mimic displaying forelimb and hindlimb of the Pel’s scaly-tailed squirrel morphology, (B) top view displaying the fore claw, hind claw and scaly-tail organ interacting with the surface. (C) Overall experimental set-up to determine the slip angle and coefficient of static friction. It shows the mimic descending with motor spinning at speed *ω* to give angle *β* from the horizontal axis. (D) Snapshots of the squirrel mimic on the set-up showing the time series of the experimental set-up of the squirrel mimic with scales attached on P1500 sandpaper (12.6 ± 1 µm average surface roughness) showing the movement and contact with the foam pad.

The scaled down appendage and body lengths of the museum specimen were used to create an approximate body structure. A simple skeleton was designed in SOLIDWORKS 2022 and fabricated via laser cutting an acrylic sheet (Universal Laser System PLS6.150D). The fore claws, hind claws and the scaly-tail organ were attached to the body structure as shown in figure 3A,B. Finally, four black colour ball pins were placed on the dorsal side of the model to enable image tracking.

#### 2.2.2. Experimental set-up

The role of the scaly-tail organ in the squirrel’s sliding stability was characterized through two experiments. The first experiment tested the frictional contribution of the scaly-tail organ compared with the claws using two versions of the squirrel physical model. Version one consisted of the model with all four claws and the scaly-tail organ; the second version removed the scaly-tail organ. Each version was tested on four different substrates represented by sandpapers of grit size P1500 (12.6 ± 1 µm), P600 (25.8 ± 1 µm), P150 (90 ± 15 µm) and P60 (270 ± 10 µm) [36]. A higher number following ‘P’ represents a ‘smoother’ grit paper and the value reported in the bracket is the root-mean-square average roughness (*Rq*) in µm. The *Rq* value allowed the sandpapers to be compared with tree barks of varying roughness. Previously published literature indicates that bamboo is similar to P400 sandpaper and P40 sandpaper is similar to high-roughness tree bark [33]. Therefore, we chose sandpapers that represent barks smoother than bamboo and less rough (intermediate) than high roughness bark trees like the red spruce that is inhabited by the northern flying squirrel [37]. Such a gradation allowed us to investigate the roughness at which the scaly-tail organ engages and potentially fails.

The second experiment tested the effect of the scaly-tail organ size on substrate engagement. Instead of the squirrel model, we used a rectangular plate mounted with the scaly-tail organ. We tested five sizes of the scaly-tail organ by scaling it by the following ratios: 0.5×, 0.75×, 1.0×, 1.25× and 1.5×. These ratios resulted in scale sizes of ball tip radii of 1.28, 1.92, 2.56, 3.20 and 3.84 mm, respectively. These sizes of the scaly-tail organ were tested on a single substrate (P60), which corresponded to the substrate that resulted in the steepest perch angle that could be sustained by the squirrel physical model during the sliding experiment.

Both experiments used the same set-up consisting of a ramp with changing inclination, a substrate material and an orthogonally placed monochrome high-speed camera (AOS-Smotion-104-M with AOS 50 mm lens; figure 3C,D). At least 10 trials for each iteration of the experiment were performed, out of which some were discarded due to issues with the camera trigger and tracking (electronic supplementary material, table S3).

##### 2.2.2.1. Ramp set-up

The ramp was constructed using plywood (40 × 70 × 1 cm) with a placeholder (30 × 50 cm) in the middle for substrate placement (figure 3C). One end of the ramp was connected to a hinge on an elevated platform, and the other end was connected to a pulley through a cable. The inclination of the ramp was controlled using a servomotor (Dynamixel WC430-W150-T) connected to the pulley mechanism figure 3C. The inclination was varied from a horizontal position down to a maximum of −90^o^ at a speed of 0.229 r.p.m. Four white colour ball pins were placed along the side (thick edge) of the ramp to provide high-contrast points for motion tracking. An L-shaped plywood piece with four white colour ball pins was placed on the top left of the ramp such that the longer side of the L-shaped piece was horizontal with the ground.

##### 2.2.2.2. Experimental protocol

For each experiment, the substrate was mounted on the ramp, followed by placing the model on the substrate. The topmost point of contact of the model was near the top edge of the substrate, providing sufficient space on the ramp for it to slide freely. The experiment started from a ramp inclination of 0° and increased at a rate of −0.94 ± 0.42° s^−1^ when averaged over all trials used for analysis (*n* = 123). Simultaneously, the high-speed camera recorded the trial at 200 fps with a 2 s before and after trigger buffer. As soon as the model slipped, the camera was triggered, allowing for the precise measurement of the ramp angle at which sliding occurred along with the velocity and acceleration of the model during each slide. After five trials, the mimic including fore claws, hind claws and scaly-tail organ three-dimensional prints was replaced, and a fresh set of sandpaper was placed on the ramp.

##### 2.2.2.3. Data processing

The slip angle of the model was calculated by tracking the ball pins on the physical model/rectangular plate, ramp and the L-shaped plywood piece using the deep learning pose estimation package DeepLabCut (DLC) [38]. A Resnet50 network was trained on 40 images of the scaly squirrel model, and a second Resnet50 network was trained on 201 images of the rectangular plate with the scaly-tail organ (figure 3D). The tracking output from the DLC package consisted of the pixel coordinates of all the ball pins for the entire video duration. The pixel coordinates were calibrated using the actual measurement between two ball pins on the horizontal leg of the L-shaped piece. After calibration, each ball pin track on the squirrel model, rectangular plate and ramp was smoothed using a smoothing quintic spline, followed by taking the smoothed tracks’ first and second derivatives to calculate the two-dimensional velocity and acceleration. Each track was translated and rotated such that the origin (*x* = 0, *y* = 0) corresponded to the pin on the ramp closest to the hinge and the positive *x*-axis. The instance when the model slipped was identified by the sudden change in the distance of the ball pin on the model with respect to the origin. The sudden change corresponded to a change in the slope of the distance curve plotted with time. The frame at which slip was detected was used to calculate the ramp angle and corresponded to the model’s slip angle for the static friction calculation (*µ*_s_).

### 2.3. Coefficient of static friction calculation

**Figure 4.**
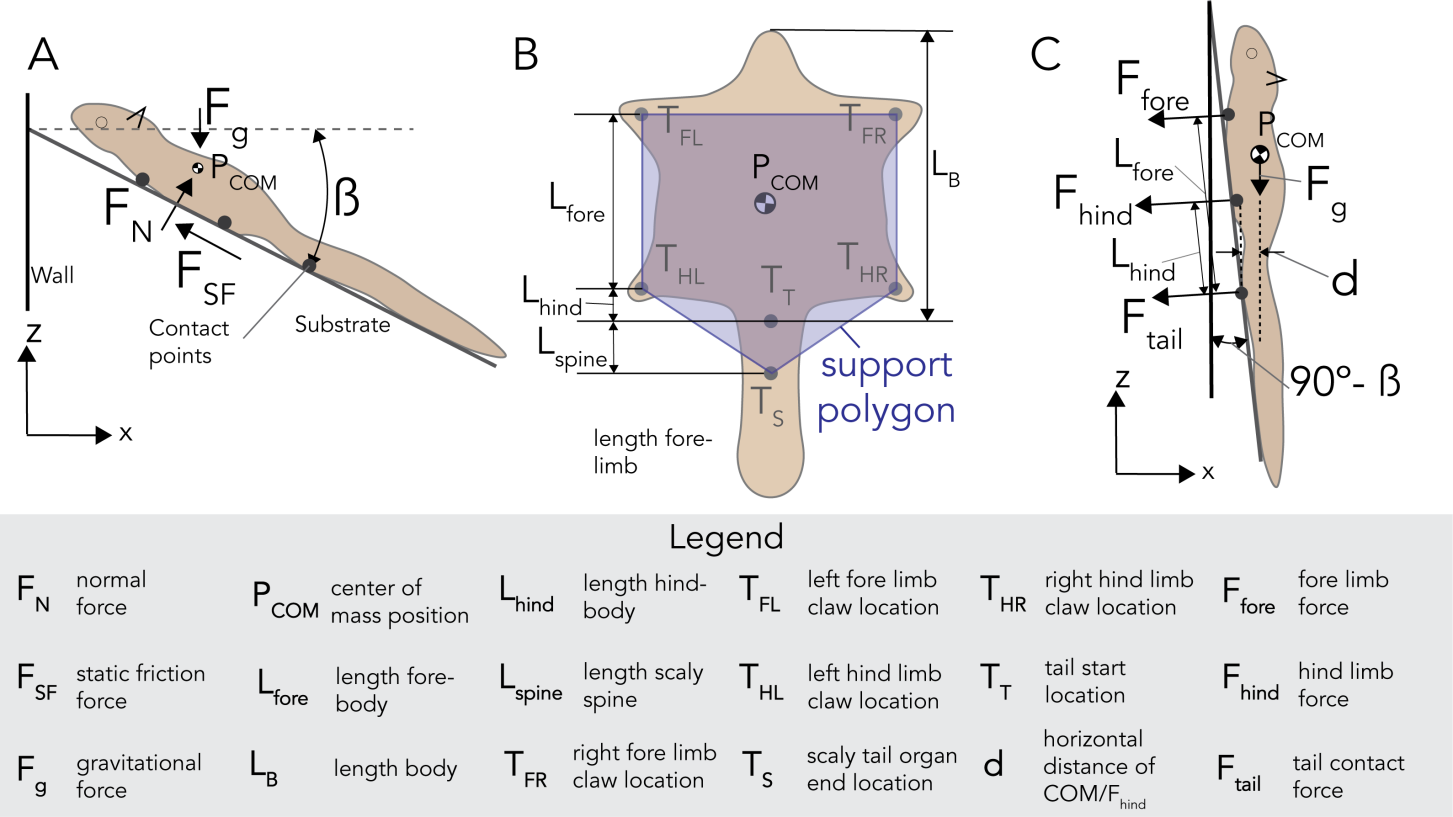
Stability of squirrel on an inclined surface. (A) Forces acting on the squirrel resting on an inclined surface, (B) support polygon formed by the four claws and the scales of the tail and (C) the force applied by the claws to resist the pitching due to the moment by the gravitational force about the tail point of contact. Legend describes all variables in free-body diagrams (A–C).


Here, we derive the coefficient of static friction equation for the squirrel model, and the same equation applies to the rectangular plate with the scaly-tail organ, as shown in Cartmill [39]. The forces acting on the squirrel when it is statically stable are the gravitational force *F*_g_, normal force *F*_N_ , and the static frictional force *F*_SF_ (figure 4A). We assume that the force of static friction increases linearly with the applied force until the maximum value is reached. Therefore, we know that the static friction force is

(2.2) $$F_{\text{SF}}\leq \mu_{\text{s}}\cdot F_{\text{N}},$$

where *µ*_s_ is the static friction coefficient between the squirrel and the tree. This value is specific to the material differences between the squirrel and the tree. Applying the first condition for equilibrium,


(2.3) $$F_{N}=F_{g}\;\cos\left(\beta \right)$$

and


(2.4) $$F_{SF}=F_{g}\;\sin\left(\beta \right).$$

Substituting the value of *F_N_* and *F_SF_* in equation (2.2), we simplify to


(2.5) $$F_{g}\;\sin(\beta )=\mu_{s}F_{g}\;\cos(\beta ).$$

Finally, we can solve this equation for the static friction coefficient *µ_s_*,

(2.6) ,$$\mu_{s}=\frac{F_{g}sin\beta }{F_{g}cos\beta }=tan|\beta |$$

where β, in this case, is the inclination of the substrate. The maximum value of *µ_s_* is tan *θ*, where *θ* is the angle at which the squirrel model starts sliding.

### 2.4. Pitching stability analysis

The utility of the tail during landing has been observed in gliding geckos landing on vertical tree trunks with a dynamic model showing that forces required at the foot would be inversely proportional to the length of the tail [15,16]. Our study focused on the static stability and hence based our model on the concept of support polygon [40]. The squirrel gets overturned when the COM projection falls outside the support polygon. In this case, the support polygon is the convex polygon formed by connecting the attachment formed by the claws (*T*_FL_, *T*_FR_, *T*_HL_ and *T*_HR_) and the scaly tail (*T*_S_), as shown in figure 4B. It is assumed that the claws and spiny tail are single support points and these points do not slide (figure 4C).

When the squirrel is perching on an inclined surface, and the projection of COM (*P*_COM_) is inside the support polygon, the squirrel is statically stable. If the inclination is higher and the projection falls outside the support polygon (figure 4C), the squirrel becomes statically unstable due to the moment generated by *F*_COM_. To avoid the pitch back of the body about the tail base (*T*_T_) as observed in climbing [14,15] and perching [16], the squirrel would have to actively apply force by using its claws to counter the moment generated by *F*_COM_. The position of *T*_S_ (i.e. *L*_spine_) influences the maximum inclination angle of the substrate that the squirrel can stand stably without actively attaching to the substrate (i.e. the inclination angle at which the projection of COM falls on *T*_S_).

The maximum inclination till the squirrel is statically stable is given by


(2.7) $$\beta_{\text{stable}}=90-{\text{tan}}^{-1}\left(\frac{H_{\text{COM}}}{L_{\text{B}}+L_{\text{spine}}-L_{\text{COM}}}\right),$$

where *H*_COM_ is the height of COM, *L*_COM_ is the distance of the COM from the snout and *L*_B_ is the body length. We computed the value of *β*_stable_ for a range of biologically possible values of *L*_spine_. The sum of moment force required to counter the moment by *F*_COM_ is given by

(2.8) ,$$2\cdot F_{\text{hind}}\cdot L_{\text{hind}}+2\cdot F_{\text{fore}}\cdot L_{\text{fore}}=F_{\text{COM}}\cdot d,$$

where *F*_hind_ and *F*_fore_ are the forces produced by the hind and fore claws, respectively. The lengths, *L*_hind_ and *L*_fore_, are the distance of hind and fore claws from the tail base, respectively, and *d* is the moment arm of *F*_COM_. We assume that the forces produced by hind claws are 0.8 times the fore claw; (*F*_fore_ = 0.8 ∗ *F*_hind_) [41], therefore, equation (2.8) can be simplified as


$$F_{\text{hind}}=\frac{F_{\text{COM}}\cdot d}{2\left(L_{\text{hind}}+0.8*L_{\text{fore}}\right)}.$$

The moment arm, d can be calculated from the inclination angle, *β* and morphological data of the squirrel

.$$d=\left(\left(\frac{H_{COM}}{tan\left(\beta \right)}\right)+L_{COM}-L_{B}\right)\cdot \;\sin\left(90-\beta \right)$$

It is clear from this equation that the position of COM can influence the force the claws need to generate. To study the influence of the position of COM on the force required by the claws to prevent pitching, we measured the *F*_claw_ for a range of H_COM_ (H_COM_ − 2*α*, H_COM_ *- α*, H_COM_, H_COM_ *+ α*, and H_COM_ + 2*α*) and L_COM_ (L_COM_ − 2*α*, L_COM_ *- α*, L_COM_, L_COM_ *+ α*, and L_COM_ + 2*α*), where *α* is 1 cm. For our analysis, we assume *P*_COM_ is 4 cm away from the chest (H_COM_) and 17 cm from the head (L_COM_). We assume this because the total body diameter of the squirrel is 10 cm, and most mammals have a higher mass closer to their chest [42]. We assume the distance of the COM from the head is near the midway of the chest cavity but is slightly skewed towards the head as the head is more massive than the tail, which is primarily hair and fur.

### 2.5. Statistical and data analysis

**Figure 5.**
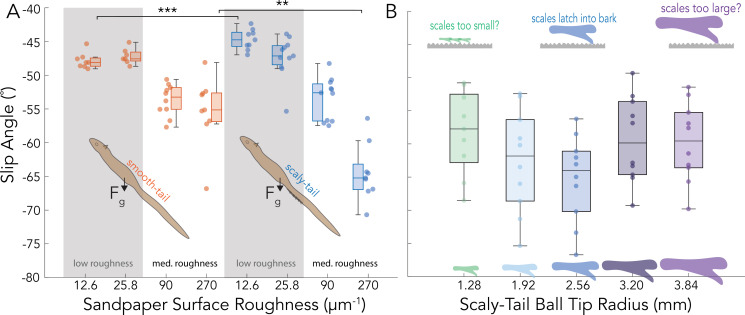
(A) Display of the slip angle *θ* of the smooth tail (orange) and scaly-tail (blue) for four different surface roughness profiles of sandpaper ranging from 12.6 to 270.1. (B) Slip angle (θ) for five different scaly-tail organ ball tip radii of curvature with insets that display the spines’ intermediate, large and small values. Statistical significance is shown with stars (^∗∗^*p <* 0.01, ^∗∗∗^*p <* 0.001).

All calculations, including statistical analysis, were performed in MATLAB 2023a (The MathWorks, Natick, MA, USA) using custom scripts (included as the electronic supplementary material). The average metrics reported throughout this manuscript are in the form of mean ± s.d., and are averaged across all trials for each substrate (figure 5A) or per spine size (figure 5B). The test for significance between with and without the scales on the same substrate was performed using the Wilcoxon rank sum test for unequal sample size. The test for significance for different scale sizes on the same substrate was performed using the Bonferroni method for multiple comparisons. The morphological data used are taken from the measurements presented in electronic supplementary material, table S3.

## 3. Results

### 3.1. Morphological data

Morphological data of the Pel’s scaly-tailed squirrel specimen, shown in figure 2A and summarized in electronic supplementary material, table S2, included gross morphological measurements of the body, claw geometry (figure 2B,C) and the scaly-tail (figure 2D). The squirrel’s body length was 37.2 cm (figure 4B). The tail length was 25.8 cm. The tail began with a short hairy portion, 1.7 cm long, which is the vertical distance between the hindlimb and the start of the tail’s scaly organ. The scaly organ was 6.9 cm long, approximately one-quarter of the tail length, similar to that of *A. derbianus* [21]. There were two rows of seven spines along the left and right sides of the scaly-tail organ, with bare skin present between each duo of scales (electronic supplementary material, figure S2). The scales’ size, area and height gradually decreased distally towards the tip of the tail (figure 2A,D). The length and width of the scales decreased distally from 7.5 × 8.5 mm to 3.2 × 3.3 mm; the aspect ratio (*L*/*W*) remained similar for all the scales. The area of the scales varied from 47.3 mm^2^ at the proximal end to 5.6 mm^2^ at the distal end. The scale height decreased distally from 6.4 mm at the proximal end to 2.6 mm at the distal end. For the limbs, the Pel’s scaly-tailed squirrel had four digits on its forelimbs and five digits on its hindlimbs.

In comparing the three friction-enhancing structures, we saw significant differences in the curvature of the point of the fore claws, hind claws and caudal organs. Using equation (2.1), we found the curvature in mm^−1^ to be 0.71, 0.54 and 0.39 for the fore claw tip, hind claw tip and scaly spine tip, respectively (figure 2B–D). The exact three-dimensional structures of these claws and the scaly-tail organ were scanned and printed (figure 2E), and all additional morphological and three-dimensionally scanned files can be found in the electronic supplementary material. Using the squirrel’s scanned morphology, we mimicked each friction-enhancing structure and tested their specific contributions to friction enhancement on diverse substrates and their contributions towards sliding stability.

### 3.2. Sliding stability

#### 3.2.1. Substrate engagement and coefficient of static friction

The slip angle (*θ*) for the squirrel model (with and without the scaly-tail organ) on each substrate is shown in figure 5A along with the corresponding coefficient of static friction (*µ_s_*) in table 1. The smooth tail model sustained approximately 14% steeper perch angles (*θ* = −54.4 ± 3.9°, *n* = 19) on the medium roughness substrate than on the low roughness substrate (*θ* = −47.5 ± 1.8°, *n* = 15). The model with the scaly-tail organ performed poorly on the low roughness substrate with *θ* even shallower than the smooth tail.

**Table 1.** The coefficient of static friction *µ_s_* for each substrate with (*s*_on_) and without (*s*_off_) the scaly-tailed organ. The substrate with a surface roughness of 270.1 µm showed a significant increase in substrate engagement with the scaly-tail organ.

roughness (µ*m*)	model type	*n*	*µ_s_*	*p*‐value
12.6	*s*_off_	8	1.11 ± 0.04	*p* < 0.001
	*s*_on_	9	0.99 ± 0.05	
25.8	*s*_off_	7	1.08 ± 0.04	*p* ≈ 0.66
	*s*_on_	11	1.09 ± 0.13	
100.1	*s*_off_	10	1.37 ± 0.12	*p* ≈ 1.28
	*s*_on_	11	1.36 ± 0.16	
270.1	*s*_off_	9	1.48 ± 0.35	*p* < 0.01
	*s*_on_	9	2.15 ± 0.39	

The smooth and scaly-tail model had significantly higher substrate engagement on the medium roughness substrate compared with the low roughness substrate, as shown by the increase in the observed *θ* and *µ_s_*. Furthermore, the substrate engagement of the scaly-tail model drastically increased on the 270.1 µm roughness, resulting in a *θ* of −64.5 ± 4.2° (*n* = 9), an approximately 21% jump in the perch steepness that could be sustained passively relative to the approximately 90 µm roughness substrate. Altogether, the maximum slip angle of the scaly-tail organ was seen on the 270.1 µm substrate roughness, resulting in an increase of approximately 17% in *θ* along with an approximately 58% increase in *µ_s_* of the squirrel model as a whole.

#### 3.2.2. Scaly-tail ball tip curvature

With a significant friction enhancement demonstrated with the scaly-tail organ on the 270.1 µm^−1^ substrate, we used the same substrate to test the influence of different scaly-tail organ sizes on substrate engagement by quantifying *β* and *µ_s_* (figure 5B and table 2). Five sizes of the scaly-tail organ were tested, which included 0.5× (1.28 mm), 0.75× (1.92 mm), 1× (2.56 mm), 1.25× (3.20 mm) and 1.5× (3.84 mm). The mean slip angle varied between −58° and −65°, with the steepest angle corresponding to the 1× scaled version of the organ (figure 5B). The 1× version sustained steeper mean slip angles than the 0.5× version, resulting in an approximately 40% increase in mean *µ_s_* from 1.69 to 2.36 (table 2).

**Table 2.** The coefficient of static friction *µ_s_* for different scaled versions of the scaly-tail organ. A multiple comparison test of the means (Bonferroni method) showed no significant difference of substrate engagement across different scale sizes.

scale	*n*	*µ_s_*
0.5×	9	1.69 ± 0.46
0.75×	10	2.11 ± 0.81
1×	10	2.36 ± 1.28
1.25×	10	1.77 ± 0.47
1.5×	10	1.79 ± 0.46

Though a U-shaped trend is indicated in figure 5, the *θ* and *µ_s_* values for the 1× version were not significantly different from the 0.5×, 0.75×, 1.25× and 1.5× versions. The maximum slip angle of the scaly-tail organ was seen in the 1× version, resulting in an increase of 10% in *β* compared with the 1.25× and an increase of 3.5% compared with 0.75×.

### 3.3. Pitching stability

**Figure 6.**
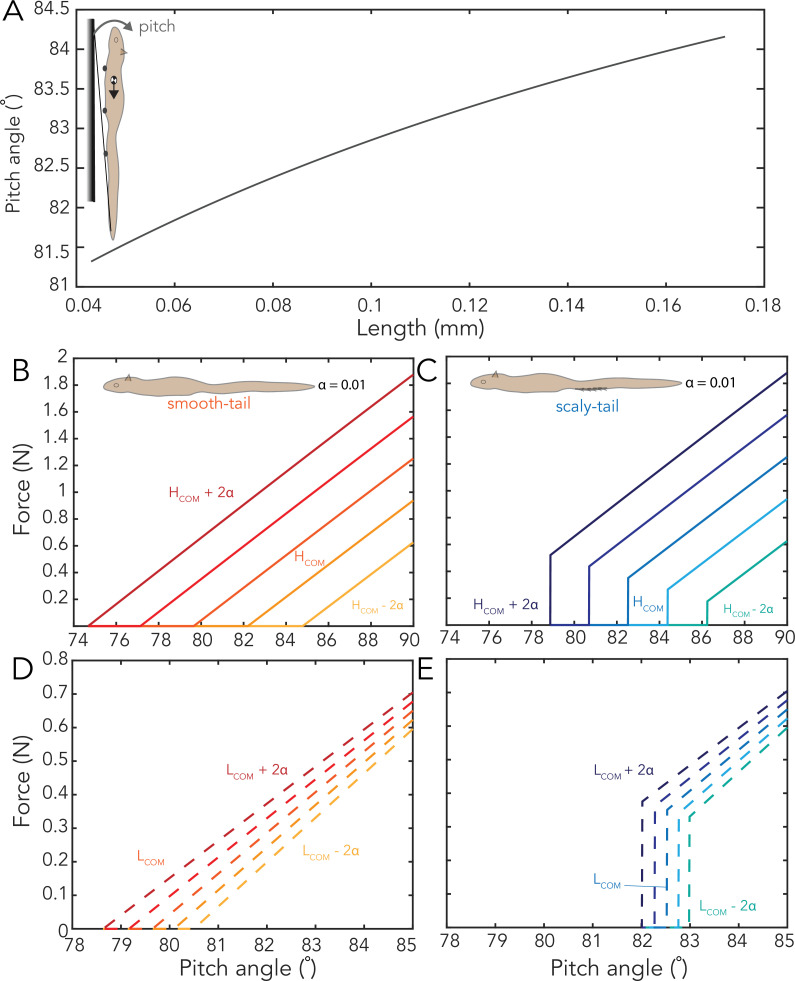
Pitching stability. (A) Variation of pitching angle for different lengths of spines and (B–D) the force required by claws to counter the pitching for different locations of COM varying the height (B,C) and varying the length (D,E).

The presence of scaly-tail changes the shape and area of the support polygon. Moreover, when the scaly-tail organ’s length increases, the support polygon’s area further increases. When the scaly-tail organ’s length was increased by 100% from the original size (8.6 cm), the maximum inclination of the substrate the squirrel can perch without applying any force by the claws is increased by 2%. However, when the tail length was decreased by 50%, the maximum inclination angle was reduced by 1.5% (figure 6A).

When the inclination increases beyond the maximum inclination angle, the claws will have to actively apply additional force to prevent the overturn. From our model, a scaly-tailed squirrel with COM position at *H*_COM_ = 4 cm and *L*_COM_ = 17 cm can reach up to an inclination of 82.5° without applying any force; however, without the scales, the maximum inclination angle is reduced to 79.6° (figure 6B,C).

We also analysed the influence of the position of COM on the additional force required by the claws. Figure 6B–E shows the force needed for the claws to grab the substrate for different locations of COM. Our model also suggests that an increase in the height of COM results in an increase in the force required by the claws. For example, at a very steep inclination of 88°, the force required by a hind claw increased from 0.39 to 1.64 N when the height of the COM increased from 2 to 6 cm (figure 6B,C). Similarly, at 84° of inclination, when the distance of COM from the snout increased from 15 to 19 cm, the force required by the claws increased from 0.46 to 0.59 N (figure 6D,E). The results show that the variation in the height of COM is more sensitive to the force required from the claws.

## 4. Discussion

The scaly-tail organ is a unique structure found at the tail base in the family Anomaluridae. The organ consists of multiple scales extruding from the tail bone, which can provide an additional mechanism to physically engage with the substrate along with the fore and hind claws. Based on this unique tail structure and the presence of ‘smoother’ bark trees in the scaly-tail squirrel’s habitat, we tested the hypothesis of the scaly-tail organ providing additional frictional enhancement (sliding stability) on substrates smoother than rough tree barks and allowing the squirrel to sustain steeper perch angles without pitching (overturning stability).

Our results supported the hypothesis that the scaly-tail organ provided additional frictional benefits on intermediate rough tree barks but performed poorly on extremely smooth surfaces. Additionally, we found that the scales may be adapted to potentially maximize engagement on intermediate roughness surfaces that resemble the tree barks found in their natural habitat. Finally, we made a simple two-dimensional squirrel model with the claws and the scaly-tail organ as contact points forming a support polygon. The two-dimensional squirrel model showed that the scaly-tail organ can allow the squirrel to sustain steeper perch angles without actively engaging the claws.

### 4.1. Scales could be adapted for ‘smoother’ barked trees

The species examined in this study, Pel’s scaly-tailed flying squirrel, is geographically located in the southwest of Ghana and the southeast of Liberia. This is primarily located in the upper Guinea rainforest zone [43], which is a unique tropical zone with a large population of drought-tolerant tree species [44–46]. The drought-tolerant species, such as *Milicia excelsa,* have a smoother bark and are inhabited by the Pel’s scaly-tailed squirrel (figure 1D). Additionally, the Pel’s scaly-tail and the Beecroft scaly-tailed squirrel have been observed to prefer habitats with abundant wild palms that tend to have smoother barks [47]. Previously, it has been hypothesized that the scaly-tail organ could be an adaptation of the squirrel’s arboreal lifestyle on trees, but no previous studies have connected the scales to smoother bark trees found in their drought-adapted rainforest habitat. Our results show that the scaly-tail organ can enhance the static frictional force on an intermediate roughness substrate that falls between a bamboo-like extremely smooth substrate and high roughness tree barks (figure 5A). For the squirrel, this frictional enhancement could lead to less slipping and reduce the energetic cost of actively engaging with the substrate for elongated periods of holding onto a perch. However, no frictional enhancement was observed on smooth substrates where the squirrel physical model with and without the scaly-tail organ resulted in similar slip angles, suggesting that the scale size was probably too large to engage with the smooth substrate.

Varying the scaly-tail organ size showed that the actual scale size (1× version) had the maximum engagement, resulting in an approximately 40% increase in mean *µ_s_* from 1.69 to 2.36 compared with the scaled-down 0.5× version. It should be noted that the 1.0× version did not show a significant difference compared with the 0.5×, 0.75×, 1.25× and 1.5× versions. Nonetheless, the significant enhancement in static friction on the intermediate roughness substrate, combined with the U-shaped slip angle trend from the 0.5× to the 1.5× version (figure 5B), suggests that the scaly-tail organ could be an adaptation to enhance the perching capabilities of the scaly-tailed squirrel on the ‘smooth’ barks that are found in their natural habitat. By enhancing the interaction with the arboreal substrate, adaptations such as the scaly-tail organ can provide instantaneous mechanical feedback and compensate for the loss in frictional forces from unforeseen perturbations experienced by the animal.

Comparing the limited morphology data available on these elusive scaly-tailed squirrels, we see that the Pel’s scaly-tailed squirrel had the longest scaly-tail organ of 69 mm compared with *A. beecrofti*, *A. derbianus* and *A. pusillus* that had lengths of 40, 64 and 34 mm, respectively [16]. It is possible that as the Pel’s scaly-tailed squirrel has the longest scaly-tail organ, this allows for the most robust attachment as in other species like geckos, where larger size and area allow for a higher maximum force [48]. Overall, while our mechanical and simulation tests demonstrate potential benefits of the scaly-tail organ on certain substrates, field studies documenting actual habitat selection and behavioural patterns are needed to establish definitive links between this morphological feature and ecological specialization. Future studies should compare the scaly-tail organ engagement across species to identify if the organ size and shape are adapted to the tree type or vary based on the species body size.

### 4.2. Contribution of scaly-tail organ towards pitching stability

The presence of friction enhancement features to scale arboreal substrates is common in the animal kingdom [6]. Numerous studies on lizards have shown the advantages of claws for clinging and interacting with various substrates [8,49,50]. Interestingly, the scaly-tail organ not only provides frictional enhancements similar to the fore and hind claw, but its anatomical location can act as a fifth point of contact, enlarging the area within which the COM lies during hanging and vertical locomotion [15,16]. This fifth point of contact could help offset the fact that larger animals often attach less well to surfaces [6]. The area formed, referred to as a support polygon, can allow the squirrel to maintain passive stability while hanging without risking pitching and falling to the ground. Our two-dimensional mathematical model for pitching stability showed that the scaly-tail organ can enable the squirrel to sustain an approximately 4% steeper perch angle passively compared with a four-point support polygon formed by the fore and hind claws only. Since the steeper perch angle is a consequence of passive perching, this can significantly reduce the energetic costs of perching for the scaly-tailed squirrel on ‘smooth’ tree barks without applying additional force through their claws to prevent pitching. Such biomechanical advantages are particularly critical for animals that are primarily arboreal, especially on tree barks that provide fewer opportunities for stable attachment points through claws alone. Overall, employing the scaly-tail organ during perching reduces the possibility of pitching back potentially conferring a selective advantage by reducing the risk of accidental falls, enhancing energy efficiency and facilitating critical behaviours such as foraging and predator vigilance. However, this hypothesis remains to be rigorously tested through further comparative ecological and biomechanical studies.

### 4.3. Bio-inspired friction enhancement for perching

Arboreal environments are challenging to explore and collect data [51,52]. Robotics engineers have focused on two strategies to explore arboreal environments: climbing robots [14,15] and aerial robots [15,16,53]. In both these approaches, the robots often must perch on the tree trunks and branches to collect the environmental data. Perching allows the robots to depend less on their locomotion modes to stay stationary, resulting in less control effort and extended battery life. Therefore, perching extends the total mission time of the robots during their operation in challenging environments like the thick canopies of arboreal environments. Various methods have been introduced so far to address the perching challenges, such as gecko-inspired adhesives, tails, mechanical spines and bird-inspired claws [16,54–57].

Our study on the scaly-tailed squirrel’s caudal scaly-tail organ provides valuable insights for innovative robot perching mechanisms. By incorporating these insights, we can introduce a novel design that enhances the sliding and pitching stability of the robot during perching. One approach involves integrating additional support points through passive spines on the chassis of the robot. These spines could mimic the functionality of the squirrel’s caudal scaly-tail organ, enabling the robot to securely grip onto branches and tree trunks. Spines enhance the sliding stability, by increasing the frictional resistance, and improve pitching stability [15,16], by increasing the area of the support polygon, as the spines act as an additional support point.

The implementation of spines on additional appendages with morphing/retractable capabilities [58,59] could offer further versatility in perching. These appendages can adapt to varying surfaces and angles, providing the robot with enhanced stability on diverse surfaces. Furthermore, customizing the morphology of the scales to match the nature of the trees in the arboreal environment where the robot operates is also crucial. Potentially, optimizing the angle of the claws could assist with friction enhancement as it has been modelled in small vertebrates [60]. By selecting scale structures tailored to the roughness of the bark through morphing capability or retractable mechanisms, the robot can optimize its stability, thereby improving overall performance and mission success in challenging arboreal environments.

### 4.4. Study limitations

Scaly-tailed squirrels are one of the least studied mammalian clades [21], which could be because of their arboreal and nocturnal lifestyle and their presence in a small geographical region. Given how understudied these species are, we had limited access to museum specimens, limiting our morphological data to a single mounted skin. This limitation of single mounted skin also did not allow us to compare the hindlimb to the forelimb for muscle density. Therefore, we used the comparison of limb length to estimate force differences between the forelimbs and hindlimbs.

Moreover, we could not observe live animals in the wild and do not know to what degree scaly-tailed squirrels actively control their compliant tail. Therefore, we assumed the tail and body are rigidly connected. This assumption is justified as our study focuses only on the role of the scaly-tail organ in the perching stability of the squirrel. The role of compliance of the body and the tail in the perching stability is an important area that can be explored in future studies.

In our experiments, we could not do mechanical frictional testing, such as in [15], on any native trees for Pel’s scaly-tailed squirrel. In future studies of the scaly-tailed squirrel, it would be worthwhile to benchmark the frictional surfaces they often interact with, which has been done in many North American gliding squirrel species [37]. Additionally, the frictional comparison of bark to sandpaper was not within the scope of this study and was based on inferences drawn from a previous study [33]. Future studies should examine and incorporate the frictional properties of different rough- to smooth-like barks to better understand the interaction between the scaly-tail organ and the substrate.

All analyses in this study model the substrate as flat, though the tree trunks the animal interacts with are curved. This curvature allows a perching animal’s limbs to subtend a central angle [61], enabling adducting forces that increase contact force at the forelimbs and hindlimbs. Since overturning torques typically act around the hindlimbs or tail contact points, arboreal animals often have longer forelimbs than hindlimbs to maximize adduction forces [39,61], which might be the reason why *Anomalurus* forelimbs are similarly elongated. These forces enhance friction at contact points, potentially affecting the role of the scaly-tail organ in preventing sliding and pitching—an aspect that could be incorporated into our analyses. We chose to test both the physical model and the simulation on flat surfaces for two reasons: first, to simplify the analysis, and second, to consider it as an extreme case, representing the most challenging scenario for the squirrel. Thus, flat surfaces serve as a conservative test substrate for assessing stability mechanisms. Finally, our physical model is designed as a rigid body system with no movable parts with a view to fixing the COM. However, depending on the pose and orientation of live animals during perching, segments can be under strain, leading to deformations and thus changes in COM, altering climbing and clinging mechanics [62]. Therefore, future studies should empirically address the perching behaviour of scaly-tail squirrels to better inform the potential role of the scaly-tail organ during perching in these animals.

## 5. Conclusion

Our study provides novel insights into the scaly-tail organ’s contribution towards enhancing the pitch stability during perching of the scaly-tailed flying squirrel in its natural habitat. Our results suggest that the scaly-tail organ could be an adaptation for the squirrel to increase friction and reduce energetic costs while perching in their ‘smooth’-bark tree habitat. Using printed mimics from the three-dimensional scans, we demonstrated that the scaly-tail organ could help reduce skid and provide significant frictional benefits on surfaces of similar roughness to trees in its natural habitat. Additionally, our findings show that the scale size is probably adapted to maximize engagement [15] with intermediate roughness substrates. Finally, through simulation, we showed that the scaly-tail organ enhances the squirrel’s pitching stability by acting as an additional contact point.

Our findings highlight the importance of using physical models to understand the complex mechanics of climbing adaptations in species that are challenging to study. To the best of our knowledge, this is the first study on these unique arboreal rodents that provides support for the role of the scaly-tail organ to enhance holding and perching stability, especially in the context of their natural arboreal environment. Altogether, our study provides a deeper understanding of the environmental stresses that might have driven the adaptation of the scaly-tail organ and identifies aspects of the tail morphology that could be translated to robot design to improve their locomotion capability on inclined and vertical substrates.

## Data Availability

All data will be accessible in an Edmond repository of MPG [63]. The museum specimen’s body measurements and scaly-tail organ measurements are included in the electronic supplementary file. Supplementary material is available online [64].
